# Brain and Mind Integration: Childhood Sexual Abuse Survivors Experiencing Hyperbaric Oxygen Treatment and Psychotherapy Concurrently

**DOI:** 10.3389/fpsyg.2018.02535

**Published:** 2018-12-14

**Authors:** Rachel Lev-Wiesel, Yair Bechor, Shir Daphna-Tekoah, Amir Hadanny, Shai Efrati

**Affiliations:** ^1^The School of Creative Arts Therapies, University of Haifa, Haifa, Israel; ^2^Emili Sagol Creative Arts Therapies Research Center, Mount Carmel, Haifa, Israel; ^3^The Sagol Center for Hyperbaric Medicine and Research, Assaf Harofeh Medical Center, Ramla, Israel

**Keywords:** child sexual abuse, hyperbaric oxygen treatment, psychotherapy, phenomenology, dual treatment

## Abstract

Due to evidence that traumatic experience impacts the brain, the body (concerning sensory sensitivity), and the mind, a recent study that attempted to answer the question of whether the effects of CSA can be reversed by using a multidisciplinary approach consisting of dual treatments: hyperbaric & psychotherapy, was conducted. Its results showed that in addition to improvement of brain functionality, symptoms of distress were significantly reduced. The current paper aims to present the process as experienced by the 40 female childhood sexual abuse survivor participants. Data included participants' daily journals and drawings, and participants' summaries presented verbally and written, 6-months after the study ended. A phenomenological analysis was used. Results showed three phases, the initial phase—remembering the trauma from both physical and cognitive aspects, the second phase—physiological relaxation as well as positive memories emerge; and, the third phase—bouncing back to life. The results are discussed in light of the study theoretical model and Lev-Wiesel (2015) childhood sexual abuse conceptualization.

## Introduction

Childhood sexual abuse (CSA) is a risk factor for psychological trauma that has a negative impact on brain development and functionality (Heim et al., 2013), as well as a strong predictor of lifetime psychopathology (Hall and Hall, 2011). There is evidence that the etiologic abnormalities of psychological trauma that are caused by CSA and traumatic brain injury (TBI) involve similar neurobiological pathogenies (Hadanny and Efrati, 2016). However, although the biological, psychological, and social ramifications of the trauma have been under scientific scrutiny for some time, the conventional treatment modalities that are used to help CSA trauma survivors are merely psychological (Lev-Wiesel, 2008), sometimes accompanied with pharmacological treatment.

The dual treatment that combines HBOT and psychotherapy (Hadanny et al., 2018) concurrently was the first to address both the brain areas suffering from metabolic dysfunction and the clinical psychological symptoms CSA survivors exhibit. This theoretical study model was based on evidence that traumatic experience impacts the brain (Rinne-Albers et al., 2013), the body (in terms of sensory sensitivity) and the mind (Lev-Wiesel and Markus, 2013). It attempted to answer the challenging question of whether the effects of CSA can be reversed by using a multidisciplinary approach consisting of hyperbaric oxygen treatment and psychotherapy. The prospective randomized clinical trial was conducted between July 2015 and November 2017. It consisted of 30 women with a history of CSA who fulfilled fibromyalgia diagnosis criteria (FMS) for at least 5 years prior to inclusion. Participants were randomly assigned to the treatment group, treated with 60 HBOT & psychotherapy sessions and a control/crossover group received psychotherapy. After the control period, the control/crossover group was crossed to HBOT. The measures included fibromyalgia (FMS), Posttraumatic stress disorder (PTSD), and quality of life questionnaire, and, brain function and structural imaging. The results yielded that a significant improvement in all FMS questionnaires, most domains of quality of life (SF-36) and PTSD questionnaires, was apparent following HBOT. The same significant improvements were demonstrated in the control following crossover to HBOT. Important to note, that despite the fact that the control/crossover group received psychotherapy when serving as controls, their mental and physical well-being did not improve during that phase (some even deteriorated) (Hadanny et al., 2018). Following HBOT, brain SPECT imaging demonstrated a significant increase in brain activity in the prefrontal cortex, orbital frontal cortex, and subgenual area (*p* < 0.05). Brain microstructure significant improvement was seen by MRI-DTI in the anterior thalamic radiation, left Insula and the right Thalamus.

As described above, participants underwent psychotherapy during the HBOT. While the quantitative results were summarized and published in a previous paper (Hadanny and Efrati, 2016), the present paper seeks to describe the process of dual treatment as reflected in the participants' daily journals (narratives and drawings) written during the procedure, and their verbal retrospective reflections given verbally and in writing, 6 months after the study ended.

### Literature Background

#### Childhood Sexual Abuse: Definition and Consequences

CSA defined as a sexual activity (ranging from fondling to sexual intercourse) with a minor, perpetrated by a person significantly older than the child (Hetzel and McCanne, 2005), is a unique severe traumatic event, as it denotes actual or threat of oral, anal or genital penetration of a child who cannot fully comprehend, is unable to give informed consent, and is not developmentally prepared (Fortier et al., 2009). The psychological trauma brought about by the experience of profound threat, such as CSA, leads to a longer-term syndrome that has been defined, validated, and termed PTSD in the clinical literature, and is often accompanied by devastating functional impairment. Contingent on contextual characteristics and the victim's personal and social resources, exposure to CSA usually results in a variety of other long-term, deleterious mental and health effects, such as depression and anxiety disorders (Hovens et al., 2015), posttraumatic stress disorder (Bremmer et al., 1997), dissociative disorders, memory, and recall impairments (Wolf and Nochaiski, 2013), chronic pain fibromyalgia (Springer et al., 2003), myocardial infarction, asthma, and diabetes (Gilbert et al., 2015), and intestinal complications (Lev-Wiesel and Markus, 2013).

#### CSA, Brain, Body, and Mind

There is evidence that CSA, particularly if repeated, evokes a cascade of neurohumoral and neurotransmitter effects that produce enduring deleterious alterations in brain structure and function (Frodl et al., 2010) mainly on the development of the hippocampus, amygdala, corpus callosum, cerebral cortex and cerebellar vermis (Rinne-Albers et al., 2013). This severe early stress associated with reduced synaptic numbers in the hippocampal region explains the difficulties in memory retrieval associated with the traumatic event (Tomoda et al., 2009) and dissociative symptomatology that CSA survivors tend to suffer from (Stein et al., 1997). In addition, excessive amygdaloidal activation is found to play a crucial role in the development of PTSD (Newport and Nemeroff, 2000) and major depression (Drevets et al., 2002). This heightened activation was found also to be involved in the formation and recollection of emotional memory, the learning of non-verbal motor patterns, and the triggering of fight-or-flight responses (Teicher et al., 2003). Recently, brain imaging of female survivors of CSA (Heim et al., 2013) showed that the affecting areas involved in genital sensation were reduced, as compared to women who had no history of CSA; it was suggested and found in the current recent study (Hadanny et al., 2018) that the CSA interference with the connectivity of the somatosensory regions ultimately left those regions underdeveloped from the reduced input.

#### Treatment Interventions in CSA Survivors

Most treatment modalities for trauma survivors adopt one or more of four basic therapeutic goals (Hodges and Meyers, 2010): (1) symptom relief, which may be accomplished by encouraging the client to think differently about the event, teaching the client to manage his or her aberrant behaviors, facilitating the expression of adverse effect, affirming the child's experience, and providing emotional support; (2) de-stigmatization, which may be achieved by group affirmation from other victims and the therapist's supportive stance; (3) increasing self-esteem through cognitive and interpersonal exercises and role plays; and (4) preventing future abuse by changing the victim's environment and/or behaviors and awareness.

Some treatment modalities were explicitly tailored for CSA survivors. However, follow-up studies on treatment outcomes are rare (Lamoureux et al., 2012). Those that are described in the literature, for example, trauma-focused cognitive behavioral therapy (TF- CBT) (Deblinger et al., 2011) and child-centered therapy (CCT) (Barker-Collo and Read, 2003), were found to be effective in terms of reduction of symptoms. Other treatment modalities that were developed and are used by professionals do not have clear protocols, nor have they been scientifically tested for their effectiveness (Trask et al., 2011). Nevertheless, evidence indicates that symptom reduction is often temporary (Trask et al., 2011).

To achieve persistent recovery, all aspects, psychological and physiological, must be targeted. This led Lev-Wiesel (Lev-Wiesel, 2015) to suggest and clinically implement the specific treatment of creative art psychotherapy model that aims to restore integration between body and mind. The model uses the physical expression (through art means) to process the energy stored in the body, which resets the neurological system into better balance. It also enables the appearance of conscious and unconscious meaningful symbols, which in turn encourages verbalism. This modality answers the body and mind dimensions only. It does not consider the brain dysfunction that has been found to occur. Therefore, based on the previous evidence indicating that CSA is a unique trauma resulting in a damaged brain, a wounded body, and a distressed mind, a more comprehensive approach was required, including a metabolic intervention that is capable of inducing brain neuroplasticity. Thus, healing as the final objective of the intervention means integration between the dissociative subsystems of the victim's personality, reduction or elimination of symptoms, and reconnectivity between brain areas. Since the quantitative data including the brain scanning was presented previously, we show here the emotional process of participants as reflected throughout the study in their diaries.

## Method

### Participants and Procedure

Following Approval of the Institutional Review Board of Assaf Harofeh Medical Center (202/14), a convenient sample of 30 women who had a history of child sexual abuse was recruited. All women had already underwent psychotherapy for at least a year prior to their inclusion and fulfill fibromyalgia diagnosis criteria for at least 5 years prior to their inclusion. The mean age was 45.9 (*SD* = 8.9), average level of education was 16.5 (*SD* = 3.3), 60% were married, 73.3% were employed, and, 36.7% were religious. Concerning the abuse characteristics, 56% were abused over a year during childhood, and 66.7% were abused by a family member.

After signing an informed consent form, participants underwent baseline evaluation which included medical history, physical examination, psychological interview, questionnaires and brain imaging. Included participants were randomly assigned to two groups (1:1 randomization): a *treatment group* and a control/crossover *group*. The dual treatment included HBOT and psychotherapy concurrently. participants received. HBOT is conducted in a multi-place hyperbaric chamber. According to the HBOT protocol (Efrati et al., 2013), 60 daily HBOT sessions have been administrated 5 days per week. The treatment was comprised of 90 min exposure to 100% oxygen at 2 ATA, with 5-min air breaks every 20 min.

### Qualitative Measures

As mentioned above, in addition to the quantitative measurements and brain scanning measures that were used in the study this paper is based upon, participants kept a daily journal reporting their sensations, emotions, and experience of the treatment. This data was analyzed. Also, examples of participants' treatment summaries given to authors during a group meeting 6 months after the termination of the dual treatment, are presented.

### Qualitative Data Analysis

Interpretative phenomenology involves a conversation that brings together the discourse of the participants, the questions posed by the researcher, the interpretations offered by the participants themselves, and the interpretations provided by the researcher. The data (written daily journals) were analyzed using the procedures of hermeneutic phenomenology, as outlined by van Manen (1990), namely the selective highlighting approach. Following these methodological guidelines, the search for themes or structures of the experience involved selecting and highlighting sentences or sentence clusters that stood out as essential to the experience. It is important to note that the daily journals were sent every day, via email, to the therapist who was assigned to accompany the participant during the research. Thus, it was first read by the therapists and was later reread by the researchers, as the data accumulated and the broader clusters of themes gradually emerged.

After the collection of data was completed, the following procedures were followed:

1. The participants' accounts were read and reread as a whole, in the process of hermeneutically interpreting these texts as inscriptions of lived experience, to identify relevant and significant expressions, to identify emerging themes, to note connections and then to group them thematically.
2. Following this initial reading and rereading of the journals as a whole, they were read, again, with special attention to precise phrases and changes in feelings, sensations, and cognition over time that related to the experience of participants within and outside the hyperbaric chamber. In the course of this focused reading phase, highlighted expressions were identified and clustered into recurrent themes that revolved around significant aspects of the participants' lived experience. The analysis aimed to create a comprehensive account of the themes which seemed significant to the therapeutic procedure.
3. On completion of the individual analysis, lists of themes were compared from all journals and assembled as themes within higher-order categories.
4. At the point of saturation, which became evident when there was a recurrent replication of data concerning the emerging essential themes, marginal themes were dropped, and more prominent themes, which were found to be fundamental and distinctly related to the participants' experience, were expanded.

To maintain methodological rigor, the findings were presented at various stages of the process at research forums, offered to participants for “member validation,” and discussed with colleagues, who mostly concurred with the major themes, and added valuable methodological input. The following is an example of concerns and thoughts raised by colleagues at the discussion: To what extent variables such as the age of CSA onset, use of meditation techniques prior and during the HBOT session, present age, might impact the length of each phase?

### Findings

This section is divided into two parts: the first focuses on the themes emerged from the daily journals (including drawings); the second part presents participants' summaries of the treatment experience and outcome, 6-months following termination of the dual treatment.

#### The Therapeutic Process

The experience of receiving HBOT was divided into three phases, each consisting of about 20 sessions. The first phase was characterized by acute experiences of diffuse physical pain followed by recovered negative dissociative traumatic memories. The second phase was often characterized by body relaxation, reduction of symptomatology and recall of positive memories. The third phase was characterized by physical energy, feeling of aliveness, change of time perception concerning eager anticipation to the future.

#### The First Phase of HBOT Treatment: “The Body Remembers”

In the first phase (between 1 and 20 hyperbaric oxygen treatments), participants reported bodily pain during treatment which was usually limited to the time in the oxygen chamber. Sometimes the physical pain was so severe that participants cried and felt the need to escape. This was followed by painful memories, including retrieval of forgotten or dissociated memories.

For example, one participant reported experiencing flashbacks of being sexually molested. The flashbacks during treatment sessions, often started with unbearable sexual arousal, accompanied by shortness of breath and other symptoms of panic attacks. Fragments of memories appeared, in which several of her peers were raping her. Unexpected to her, she recovered a total repressed dissociated memory of having repeatedly been molested and raped by classmates, at the age of fourteen. She wrote: “*I can't understand how I, or anyone else for that matter, could forget such experiences… I continued to function as an excellent student; I focused on my dancing… I avoided peer gatherings, rationalizing this avoidance to myself and my parents as ‘lack of interest' and ‘will to excel'*.”

Another participant reported unbearable pain in her abdomen, “*as if someone is stabbing me with a knife*.” These symptoms were followed by the surfacing of new memories of the abuse, which revealed further abuse by her mother. “*My mother took me to the shower after he [my father] abused me. She undressed me, ordered me to open my legs and then began to abuse my genitals*.” These new, vivid, coherent and detailed memories exposed her to different narratives about her mother and her childhood. These memories of abuse by her mother had not surfaced before the HBOT, nor during psychotherapy given before the dual treatment. A day before this dissociated memory emerged, she wrote “*I can't breathe, I am so physically stressed, tensed, shaking from within, as if I have a volcano in my throat that is going to burst any minute, I shut my lips so hard, I feel as if I am burning from within, but no one sees…my body responds as if in delay, tensed, anxious, I feel as if memory is stuck in my lower back…a flashback that only the body remembers…the brain does not. My poor body is shaking and not breathing…*”

Another participant wrote a song: “*As a little child wandering hungry, hoping someone will hug me as a little girl, walking as if blind, perhaps someone will see me, the little girl who is so lost, wish someone will find me the little girl, so tired, wish someone will make room for me, to put my head, just a little girl, there is no one to rely on, so there is no little girl.”*

Describing her experience during the first phase, a 54 years old participant wrote: “*I am constantly feeling that I am suffocating. Although having the oxygen mask, I wonder whether something is wrong with the mask or is it me…I suddenly saw myself in my underwear at about four years old…my brother helped me dressing up, and suddenly he laid me on the bed and shoved his penis into my throat…I could not breathe. couldn't shout either…I think I fainted…this memory shocked me because I thought his molestation started much later when I was in school.”*

Some participants created drawings during the HBOT treatment in the oxygen chamber and included it in their daily journals, while others drew after the treatment.

**Figure 1:**
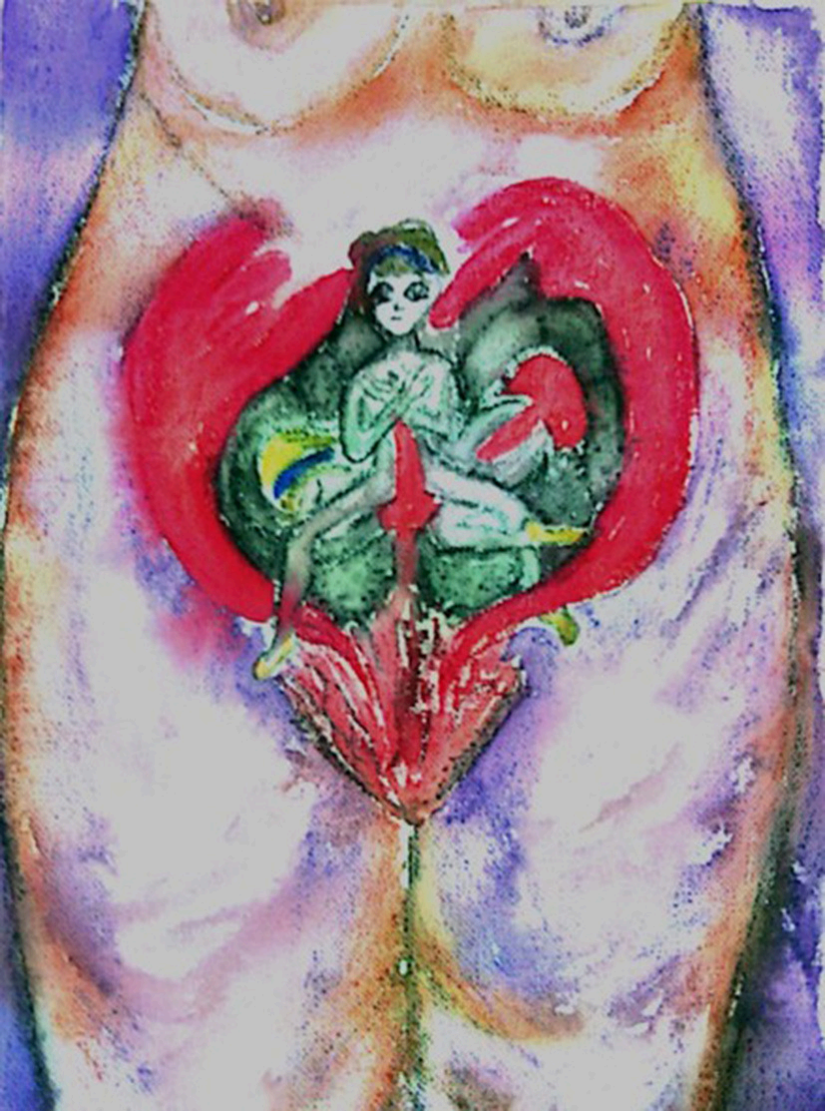
A drawing made by a 53-years-old participant, who had been sexually abused by her father.

**Figure 2:**
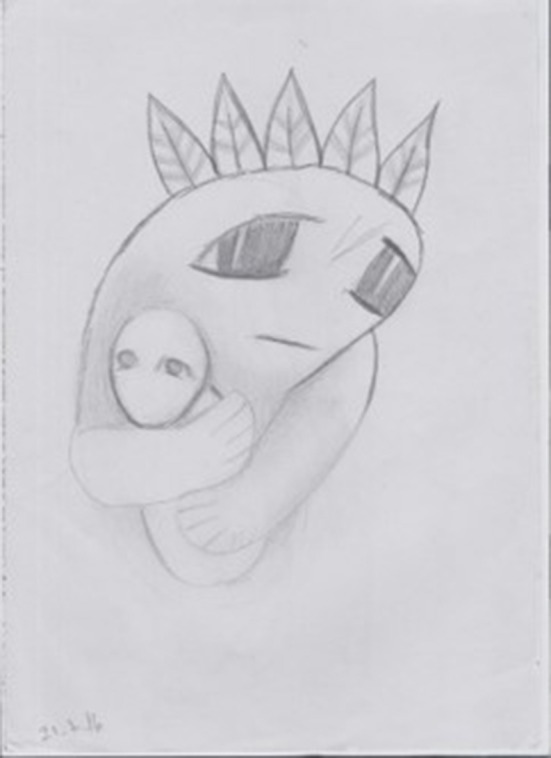
A drawing drawn during the first phase of HBOT by a 32-years-old participant, who had been sexually abused during childhood by a family member.

Here are two drawings (see Figures 1, 2) that were drawn by two participants during this first phase:

The two drawings reveal distress, fear, sadness, and entrapment. Whereas, the traumatic sexual abuse event seems apparent in the first drawing, the figure of the little girl is caught by a kind of monster in the second drawing.

#### The Second Phase of HBOT Treatment: “Relaxation and Serenity”

Participants described the second phase (between 20 and 40 HBOT sessions) as the soothing phase. Participants described the following changes: They began to feel better physically, sleeping difficulties and disturbances were significantly reduced, nightmares were gone, they felt like they had more energy during the day, and they breathed better. During the HBOT sessions, they often fell asleep, or felt as if they had been meditating or as one phrased it “*a floating state*.” As one participant wrote “*I feel as if I am floating in the chamber… as if my brain was so thirsty and now drinks and drinks…” Another wrote: “It's like I am in a safe place, resting, really resting…even if I arrive with a headache when I get out of the chamber, it's gone…*”

Regarding the painful memories, additional dimensions of observation and interaction were added to these memories, in which the study participants interacted with their younger selves, and their younger selves revealed more to them. As one participant wrote, “*A moment of intimacy with the little girl, who was left behind…I look at her, she looks back to me, revealing herself to me, dares to show how she feels…she hates him but adores him…would not be seen if he had not seen her…I saw myself back then only through his eyes…*”

During this phase physical pains vanished, participants' dissociative memories fully recovered, and feelings of sorrow and compassion for oneself increased, replacing the previous old feelings of horror, fear or disgust. Also, good positive memories emerged. For example, one participant wrote: “*My teacher in the grade 3 was smiling at me…she said in front of everybody in the class that I am a bright student and she expects me to become a scholar, which I have become…I was so proud…I never told it to my mother…wonder how I forgot about it…because it was so meaningful*.”

Another participant wrote that in spite of the enormous effort and time she had spent to participate in this research, she was happy to get into the chamber and felt so relaxed and yet energetic when the session ended, as if “*life is smiling at me*.”

**Figure 3:**
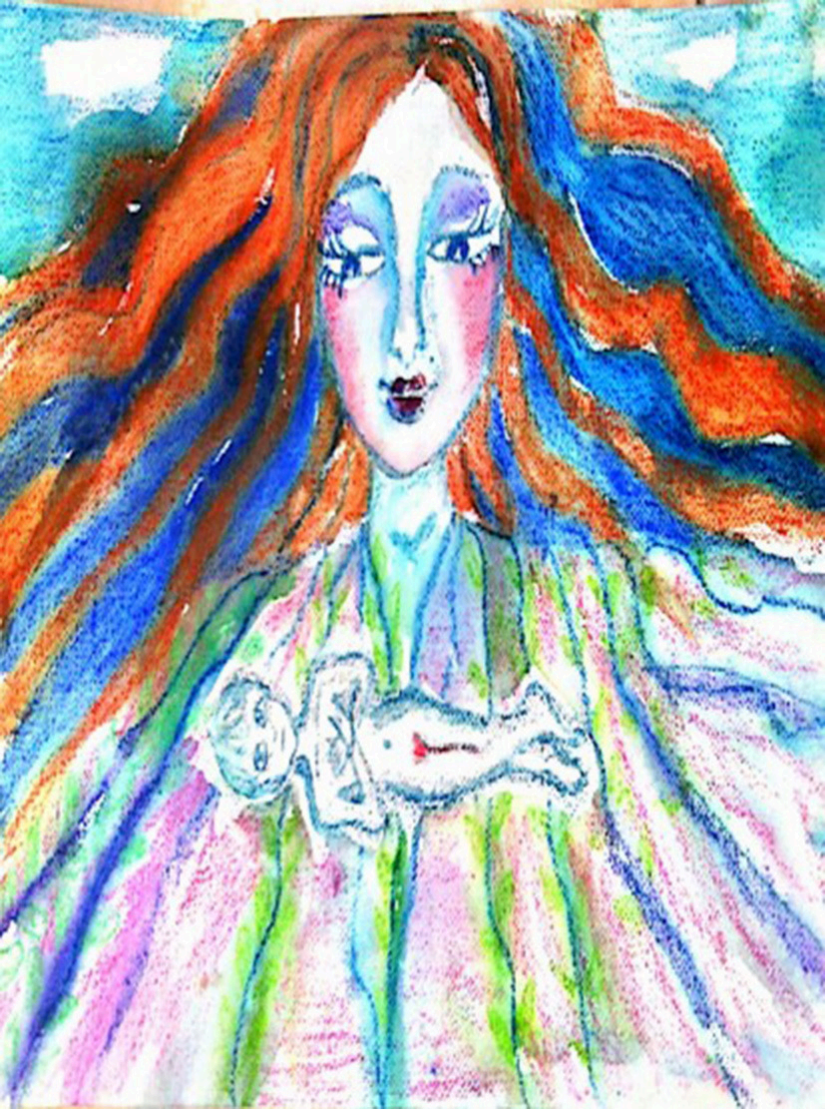
A drawing made during the second phase by a 53-years-old participant, who had been sexually abused by her father.

**Figure 4:**
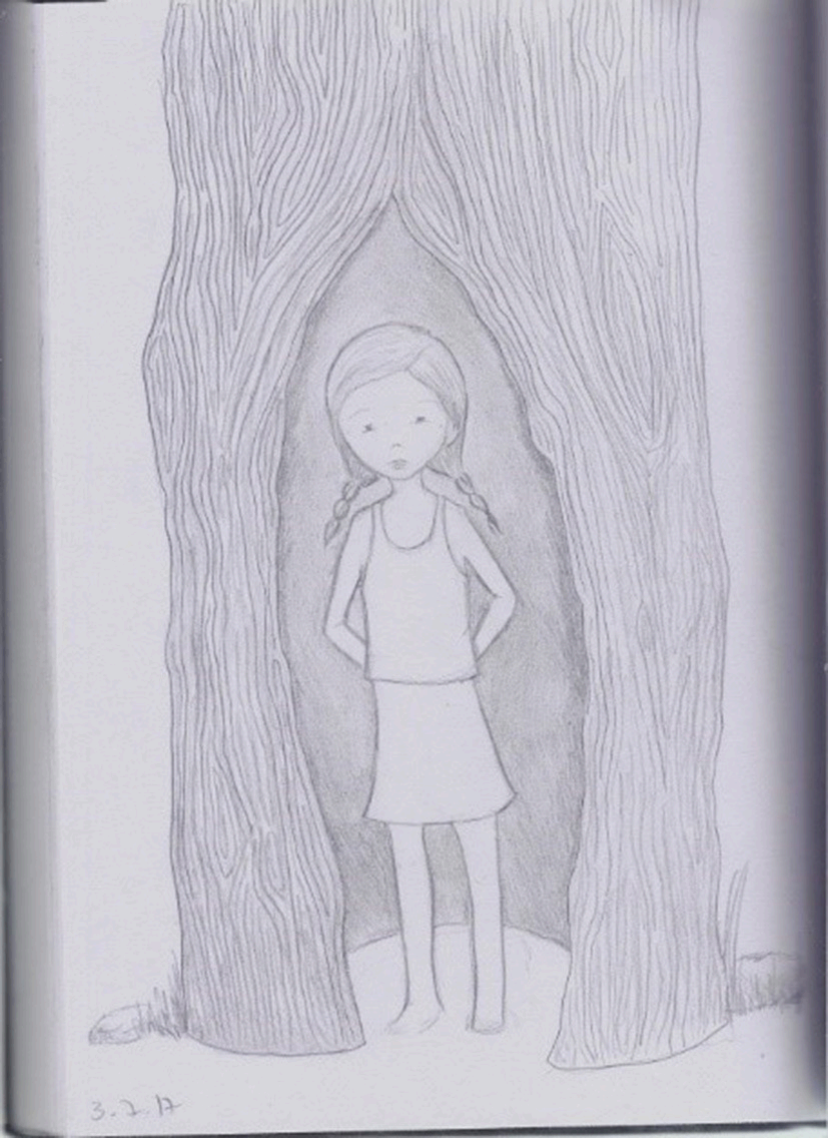
A drawing drawn during the second phase of HBOT by a 32-years old participant, who had been sexually abused during childhood by a family member.

The following are two drawings drawn during the second phase (see Figures 3, 4).

The two drawings reveal a sense of relaxation and safety. In the first drawing, the inside figure lies in the chest-stomach area as floating. In the second drawing, the girl is protected by a tree trunk yet open to the world. Although she seems not ready yet to hand her hands (hands behind her back), she can see and be seen by the world.

#### The Third Phase of HBOT Treatment: “Bouncing Back to Life”

During the third phase (between 40 and 60 sessions), participants reported a dramatic reduction of physical (Fibromyalgia) and emotional (depression, anxiety, distress, dissociation) symptoms, as well as a great increase in energy, in the ability to enjoy life, and in creativity, in the sense of coming up with new ideas of what they would like to achieve and do. They reported a change in their feelings toward the perpetrator/s and about their trauma, as well as a better ability to establish intimate relationships with people. Regarding the perpetrator and the relationship with him/her, feelings of fear and hate, or ambivalent feelings of love and hate, or of fear and longing, were replaced with indifference. As one participant wrote, “*I am not afraid anymore, it's like my past is left behind, it remains a past, it does not haunt me…I have a present and a future…the past and the people who hurt me have no hold on me anymore…so strange to feel so free.”*

Another participant described her experience during this phase as follows: “*I hear the birds, I listen to street noises, I hear myself; for the first time I notice that there are colors other than just black and white in the world… I even discovered that I have green eyes; I always thought that they were black…”*

**Figure 5:**
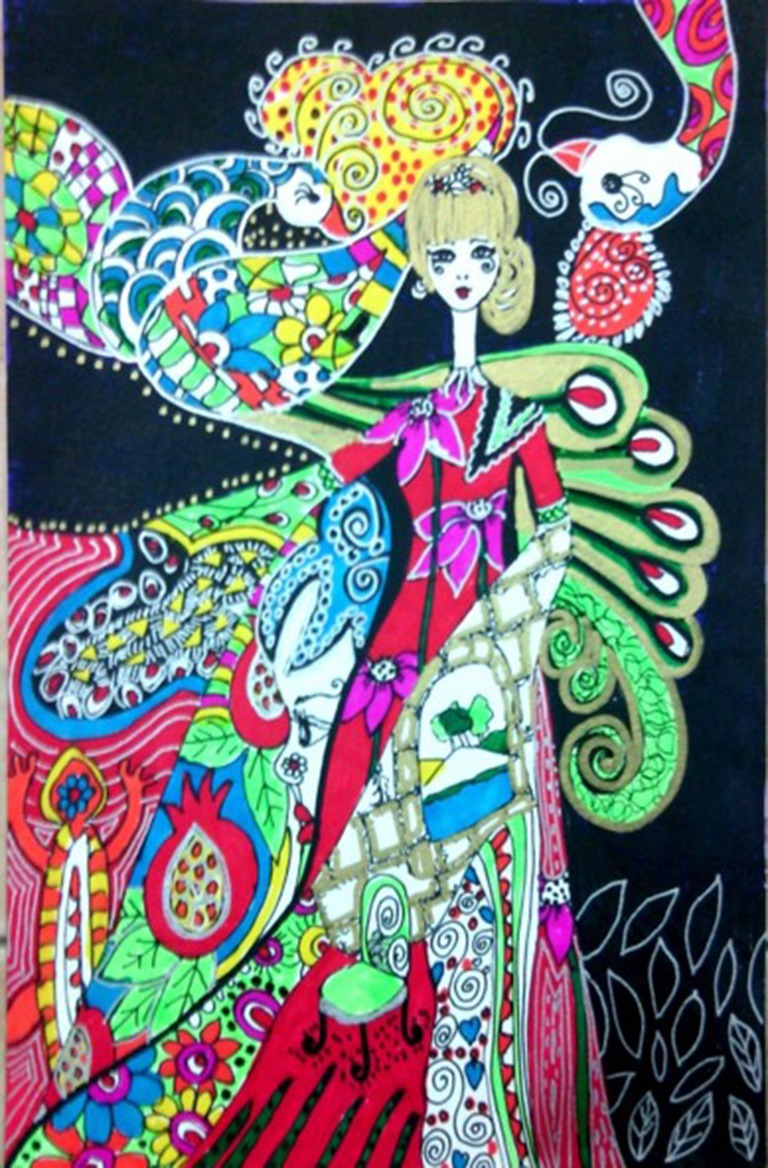
A drawing made during the third phase of HBPT, by a 53-years-old participant, who had been sexually abused by her father.

**Figure 6:**
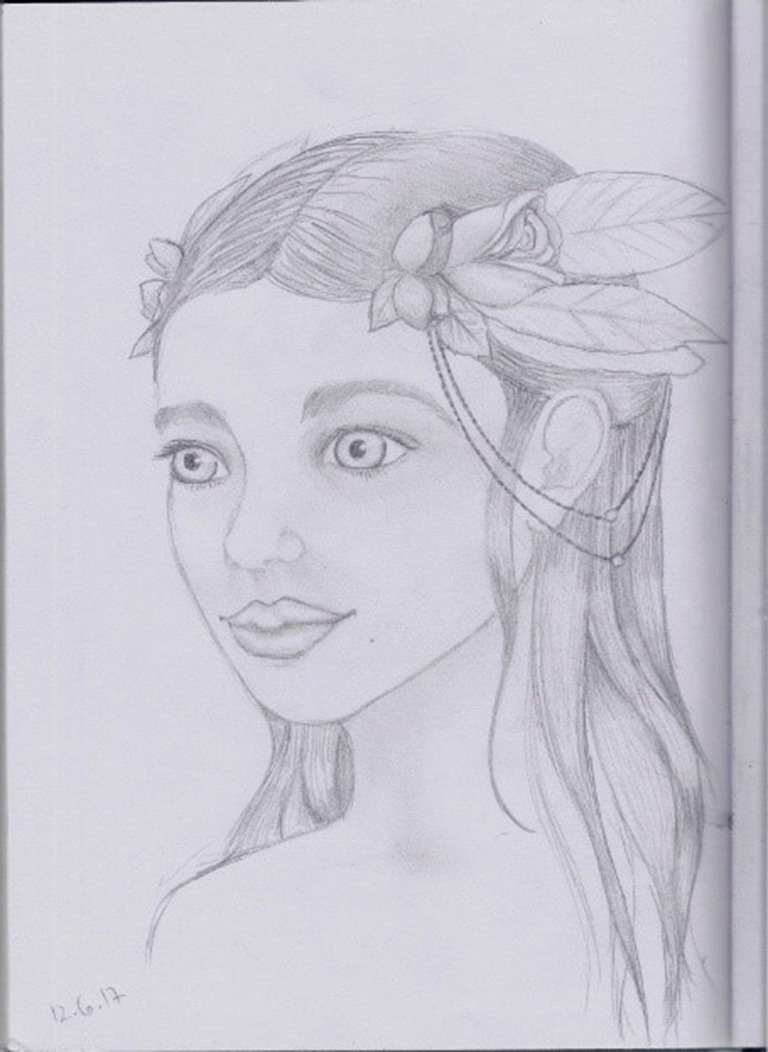
A drawing drawn during the third phase of HBOT by a 32-years old participant, who had been sexually abused during childhood by a family member.

Still another participant wrote a kind of poem: “*Today my door is closed for those who tried to hurt me, I chose, I am not surprised but rather aware of the harms and injuries, I breathe deeply into the pain, know how to soothe it, calm my soul, this is not related to the others, my wound became my own, today others can no longer hurt me, perhaps only remind me of the past hurt.”*

the following are two drawings (see Figures 5, 6) made by the same two participants whose drawings were presented in the previous two phases, during the third phase of HBOT treatment:

The figures in the drawings look happier compared to the drawings in the previous phases, facing the world smiling and not hiding in a shelter. Both look very feminine emphasizing beauty and serenity and kind of decisiveness.

### Reflecting on the Therapeutic Process and Outcome

About 6-months following the therapy completion, participants were invited to a group meeting to share their experience. They were also asked to narrate their experience. Twenty-three women attended the meeting. Five could not participate due to previous commitments, two turned down the invitation (one of these women dropped out after 50 sessions complaining of headaches worsening).

From the group meeting and the narratives submitted, it seems that the dual treatment dramatically changed participants' wellness in different life domains. One participant age 43 wrote “*The dual treatment was the shortest treatment I had ever received. I had a long history of treatments (20 years of every kind of treatment-dynamic psychotherapy, EMDR, hypnosis, mind, and body treatments, many years of meditations, etc, not even mentioning the attempts to treat myself through all sorts of opiates). Within three months of the receiving HBOT and intensive art psychotherapy, I began to feel different, gained strength, started to relate to myself as a worthiness person, a person that remember the past but not sank into it. I have a future. Believe in a better future, an optimistic future for my family and for me*.

*This wasn't always easy. There were times that I needed a lot of support from my therapist, a daily, sometimes day and night support. To overcome the vivid memories that awakened, the physical pain, the fears, the sense that the world is not a safe place- a feeling that I acquired at a very young age. I broke into uncontrolled weeping from at the beginning of the treatment during the HBOT treatment. Slowly, and with the daily accompaniment of writing and meaningful therapy sessions, the memories were cast into words and drawings, I could shed layer after layer: weightiness, depression, anxiety, helplessness, denial, and numbness. I am happy to say today that everything changed for me, how I look, the way I perceive myself, the way I cope with hardships, the life I would like to lead. I used to be afraid of losing control. I feel I gained control over myself. My movement therapy teacher, who didn't see me during the three months of treatment since I did it during the summer vacation, said surprisingly: “wow, your posture got straighter*.”

Another participant aged 54, a mother of four and grandmother to two, wrote: “*Following are the things my family had to say about the results of the treatment in their eyes, now my words seem unnecessary…E (*the husband*)- only a few months after the hyperbaric chamber treatment, V (the participant) already became an improved model of herself- more focused on the goals she wants to achieve, especially in her artistic work, she doesn't speak of her past anymore, and when it occasionally does come up, she remains calm (an extreme change for the better since before the treatment she was emotionally unstable). I feel like there are no longer screens between us, V after the treatment is no longer constantly on guard and has no separation anxiety. I always knew she loved me, but now I also feel it, powerfully. During the treatment, we had deep conversations that contributed to our closeness, because V was finally able to verbalize repressed emotions and touch places that were taboo and felt like a wall that must not be passed. The walls went down. V is freed from her past. She is happy in life and made peace with the past, while her focus is the present and the future. M (the* participant's daughter*)- she was always the most amazing mother in the world- attentive, loving, filled with the spirit of life and humor, but with it, there was also a black pit inside. A pit of loneliness and pain that as a child I always hoped I could fill and lighten. My only difficulty with my mother was her falling into that pit; then difficult emotions had arisen; emotions that I thought I did everything I could to heal, and I realized in these moments that all the good I tried to do was meaningless. It took me a long while to understand that filling this pit is not up to me and not even up to dad- that stands with mom as a single unit. In the entrance to that black pit, there were ancient snakes that didn't let the present life enter. It was filled with scorpions of the past that we couldn't kill. After grandpa passed away, mom fell deeper. It was a difficult year. A year that in every conversation with her I felt like I was stepping on eggshells. Like an ill person where every position is painful for her, I felt that no matter what I did, an arrow was shot into her heart. And then there was the hyperbaric chamber treatment. I encouraged the idea from the beginning, why not try? She might get a physical relief even if it doesn't help emotionally. The results- amazing!!! We are still faced with crises inside the house and out, but Mom no longer responds to them in an unstable manner…”*

Another participant narrated her experience retrospectively: “*I have come full circle, a chapter that began when I was a little girl comes to an end. I grew up in ‘as if' a wonderful intellectual home, which appeared to be surrounded by abundance and love. Throughout my childhood, I was exposed to sexual exploitation…I finally succeeded to break the silence. To see, understand, take in, have strength, and act against the numbing smoke-screen, social expectations and my fears, I had to use detachment, dissociate myself from myself, and keep a distance from others. The dual treatment was the shortest treatment I had ever received. I had in a long history of treatments (20 years of every kind of treatment-dynamic psychotherapy, EMDR, hypnosis, mind, and body treatments, many years of meditations, etc, not even mentioning the attempts to treat myself through all sorts of opiates). Within three months of receiving HBOT and intensive art psychotherapy, I began to feel different, gained strength, started to relate to myself as a worthy person, a person that remembers the past but does not sink into it. I have a future. I believe in a better future, an optimistic future for my family and for me. This wasn't always easy. There were times that I needed a lot of support from my therapist, daily, sometimes night and day support. To overcome the vivid memories that were awakened, the physical pain, the fears, the sense that the world was not a safe place- a feeling that I acquired at a very young age. I broke into uncontrolled weeping at the beginning of the treatment during the HBOT treatment. Slowly, and with the daily accompaniment of writing and meaningful therapy sessions, the memories were cast into words and drawings, I could shed layer after layer: weightiness, depression, anxiety, helplessness, denial, and numbness. I am happy to say today that everything changed for me, how I look, the way I perceive myself, the way I cope with hardships, the life I would like to lead. I used to be afraid of losing control; I feel I gained control over myself.”*

## Discussion

The purpose of this paper was to describe the cognitive-emotional process participants experienced during HBOT through their narratives and drawings as written in their daily journals. Also, participants' retrospective summaries of the HBOT & psychotherapy experience as presented 6-months following termination of the treatment, are presented. The qualitative findings seem to be in line with the quantitative data results and the change in brain functioning.

Before discussing the qualitative findings, it is important to restate the theoretical model for the whole study (the medical, quantitative and qualitative data). It was based on the assumption that in order to cope with an overwhelming CSA traumatic event, the brain alters patterns of signaling from the pathways involved by reducing blood perfusion and oxygen supply. This, in turn, results in a decrease in connectivity to the regions of the brain, such as the somatosensory areas. During stress, a lack of blood/oxygen perfusion results in a change from aerobic to non-aerobic brain metabolism, just like the penumbra generated after stroke or TBI. Somatosensory areas create a map of the body on the brain, with each region processing sensation from specific body parts. This interference in the level of connectivity between the somatosensory areas and prefrontal areas exhibited in a high level of dissociation enables the split between the victim's body and mind, which enhances the individual's chance for survival (Nijenhuis and van der Hart, 2011). The division of the dissociative parts of the personality manifested in symptoms that can be categorized as negative (functional losses such as amnesia and paralysis), positive (intrusions such as flashbacks), and psychoform (symptoms such as amnesia, trance episodes), or somatoform (symptoms such as anesthesia or tics) (Van der Hart et al., 2006). In combination with exaggerated amygdalar responses seen in CSA survivors with PTSD, a limited capacity for discerning threat due to hippocampal and amygdalar dysfunction may promote hypervigilance, behavioral activation, exaggerated stress responses, and further acquisition of fear associations. The disrupted prefrontal cortical function may then serve to facilitate further mental and health symptomatology, such as memory and recall impairments, emotional distress, anxiety, self-injurious behaviors, eating disorders, addiction, and chronic pain syndrome, as a result of deficient suppression of stress responses, fear associations, and extinction.

As we found, brain neuroplasticity can be induced and brain function can be restored by hyperbaric oxygen treatment (HBOT), which in turn increase the connectivity between the affected areas of the brain. As a result, a decrease of dissociation level and symptoms on the one hand, and better cognitive and emotional coping abilities as well as awareness, on the other hand, were exhibited. This process could be vividly seen in the three phases of HBOT as described by the participants. At the first phase, the body seemed to recall the painful physical sensations, followed by cognitive and emotional negative memories that were until than dissociated and forgotten. At the second phase, following the reduction of symptoms of distress such as nightmares, hypervigilance, anxiety and depression, a physical and emotional relaxation state, took over. The third phase was characterized with new physical and emotional energy, life enthusiasm, and most importantly, a change in time perception; participants seemed to move from past orientation to future orientation. The past no longer shadowed the present, allowing the survivor to experience the present and thrive toward a new exciting free of past burdens, future.

The results of this study seem to support Lev-Wiesel and Amir (2014) and Lev-Wiesel (2015) recent conceptualization of childhood sexual abuse. It posits the following five traumagenic constructs: mind/soul's homelessness—the split between the body and mind, captured in time—the present and future as reflections of the past, entrapped in distorted intimacy—lack of authenticity, betrayal entrapment—the all in all betrayal, and re-enactment—the need to relive the experience. From a clinical therapeutic view (Lev-Wiesel, 2015), a split between the mind and body occurs (Van der Kolk, 2000) since the child can no longer perceive the body as “safe home.” Escaping the abusive situation can be often possible only virtually in the victim's mind, whereas the body continues to endure suffering (Silberg, 2014). In an attempt not to feel, not to hear, not to see, not to be, dissociation is activated, dividing the child's personality (the dynamic, biopsychosocial system as a whole) into dissociative subsystems that are insufficiently integrated dynamic but excessively stable (Nijenhuis and van der Hart, 2011). This results in a split between the child's body and mind, which determines the victimized child's characteristic mental and behavioral actions, and induces symptoms such as self-injurious behaviors, eating disorders, and even suicidal thoughts and attempts, as researchers suggested (Lev-Wiesel and Amir, 2014). Indeed previous studies indicated that adult survivors of CSA show high levels of persisting dissociation (exhibiting in confusion and disorientation, amnestic episodes, nightmares, flashbacks, de-personalization, de-realization, feelings of not belonging, and detachment) compared to others with no history of CSA (Lev-Wiesel and Daphna-Tekoah, 2010).

The findings lead to a new understanding of treatment; if healing and “bouncing back to life” rather than merely reducing symptoms, is the ultimate goal of treatment, than the wounded brain and the damaged hurt self, due to the psychological trauma, should be treated simultaneously. It is an intensive therapeutic intervention which demands 3 months of total dedication to the process by both the survivor and the therapist. However, as presented by the participants of this study and summarized by another: “it was worth big time…If I had not experienced this dual treatment, I would never have believed it will make such a change in my life…I wish all CSA survivors could receive this treatment…”

## Ethics Statement

This study was carried out in accordance with the recommendations of The Helsinki committee with written informed consent from all subjects. All subjects gave written informed consent in accordance with the Declaration of Helsinki. The protocol was approved by the Asaf Harofe Helsinki committee.

## Author Contributions

SE and AH were responsible for the hyperbaric oxygen therapy sessions and analyzing the quantitative data. RL-W with YB and SD-T were responsible for recruitment, collecting and analyzing the qualitative data, and provide psychotherapy to participants. RL-W was responsible to write the final report.

### Conflict of Interest Statement

The authors declare that the research was conducted in the absence of any commercial or financial relationships that could be construed as a potential conflict of interest.
